# Continuing Threat of Influenza (H5N1) Virus Circulation in Egypt

**DOI:** 10.3201/eid1712.110683

**Published:** 2011-12

**Authors:** Ghazi Kayali, Rabeh El-Shesheny, Mohamed A. Kutkat, Ahmed M. Kandeil, Ahmed Mostafa, Mariette F. Ducatez, Pamela P. McKenzie, Elena A. Govorkova, Mohamed H. Nasraa, Robert G. Webster, Richard J. Webby, Mohamed A. Ali

**Affiliations:** St. Jude Children's Research Hospital, Memphis, Tennessee, USA (G. Kayali, M.F. Ducatez, P.P. McKenzie, E.A. Govorkova, R.G. Webster, R.J. Webby);; National Research Center, Giza, Egypt (R. El-Shesheny, M.A. Kutkat, A.M. Kandeil, A. Mostafa, M.H. Nasraa, M.A. Ali)

**Keywords:** Avian influenza, H5N1 virus, surveillance, viruses, influenza, Egypt

## Abstract

Reservoirs for the continuing influenza (H5N1) outbreaks in Egypt are ill-defined. Through active surveillance, we detected highly pathogenic influenza subtype H5 viruses in all poultry sectors; incidence was 5%. No other subtypes were found. Continued circulation of influenza (H5N1) viruses in various regions and poultry sectors perpetuates human exposure in Egypt.

After 150 confirmed human cases and continuous outbreaks in its different poultry production sectors, Egypt became an epicenter for highly pathogenic avian influenza (H5N1) virus activity and one of the few countries where this virus is endemic. The long-term endemicity of influenza (H5N1) virus in poultry in Egypt has generated substantial viral genetic and antigenic diversity, as has been seen in other areas (*1**–**3*), yet the ecology and epizootology of the virus in the various poultry sectors remains unknown. To determine the incidence and diversity of influenza viruses among poultry in 6 governorates in Egypt, we conducted surveillance for 1 year.

## The Study

From August 2009 through July 2010, we collected 5,562 cloacal and oro-pharyngeal swab samples from poultry at 58 sites in 6 governorates in Egypt (Cairo, 4 sites, 24 birds/100,000 inhabitants; Qalubiya, 12 sites, 317,000 birds/100,000 inhabitants; Menofiya, 9 sites, 436,000 birds/100,000 inhabitants; Sharkiya, 2 sites, 375,000 birds/100,000 inhabitants; Fayyoum, 22 sites, 98,000 birds/100,000 inhabitants; and Beni Suef, 9 sites, 108,000 birds/100,000 inhabitants) (*4*). The selected governorates represent the main foci of the poultry industry in Egypt. In each governorate, 2–6 sites were routinely sampled monthly; samples were also collected from other sites and villages in the same governorate. Sample collection, handling, transport, screening, and subtyping by reverse transcription PCR (RT-PCR) were performed according to published protocols (*5**–**7*).

Of the cloacal swab samples, 5.0% were positive for influenza (H5N1) virus by matrix gene RT-PCR; of the oropharyngeal swab samples, 4.9% were positive (Table). All positive samples contained hemagglutinin subtype H5, determined by H5-specific RT-PCR. The percentages of positive samples by governorate were 0%–13.1% (p<0.001, Pearson χ^2^ test). Positivity rates were higher for governorates in the Nile Delta region (Qalubiya, Sharkiya, and Menofiya) and Cairo (6%) than for those in southern Egypt (Beni Suef and Fayyoum, 0%–3.8%).

**Table:** Epizootologic data for avian influenza (H5N1) virus, Egypt, August 2009–July 2010*

Variable	No. (%) samples		p value†
	Collected	Positive	
Swab type			NS
Cloacal	4,353 (78.3)	217 (5.0)	
Oropharyngeal	1,209 (21.7)	59 (4.9)	
Governorate			<0.001
Cairo	979 (17.6)	58 (5.9)	
Qalubiya	916 (16.5)	120 (13.1)	
Menofiya	1,636 (29.4)	27 (1.7)	
Sharkiya	280 (5.0)	21 (7.5)	
Fayyoum	1,323 (23.8)	50 (3.8)	
Beni Suef	428 (7.7)	0	
Species			<0.001
Breeder chickens	50 (0.9)	5 (10.0)	
Broiler chickens	3,803 (68.4)	163 (4.3)	
Layer chickens	710 (12.8)	97 (13.7)	
Ducks	819 (14.7)	10 (1.2)	
Geese	55 (1.0)	0	
Pigeons	51 (0.9)	1 (2.0)	
Turkeys	74 (1.3)	0	
Location			<0.001
Abattoir	330 (5.9)	38 (11.5)	
Commercial farm	2,827 (50.8)	192 (6.8)	
Backyard flock	1,381 (24.8)	12 (0.9)	
Live-bird market	1,024 (18.4)	34 (3.3)	
Bird health status			<0.001
Healthy	5,255 (94.5)	235 (4.5)	
Sick	214 (3.8)	35 (16.4)	
Dead	93 (1.7)	6 (6.5)	

*NS, not significant. †Pearson χ^2^.

**Figure 1:**
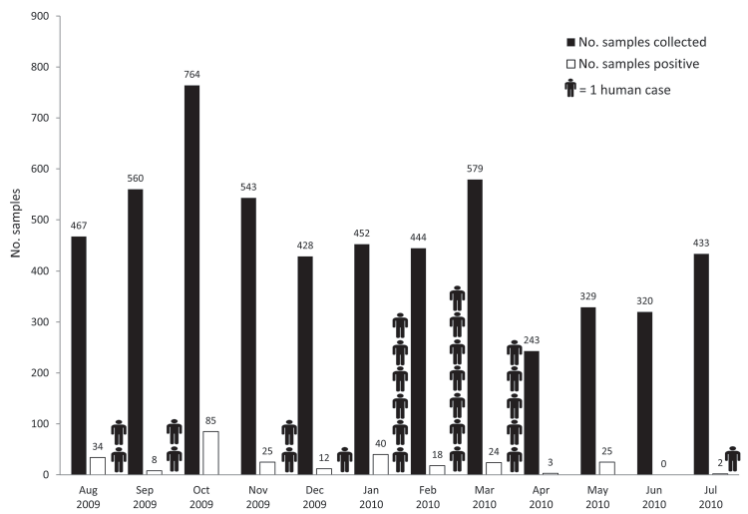
Number of samples collected from poultry and number positive for influenza (H5N1) virus, Egypt, August 2009–July 2010.

A total of 243–764 samples were collected monthly, depending on the number of poultry available at the sites during the sample collection visit (Figure 1). At least 2 samples tested positive in every surveillance month except June 2010. The positivity rate was highest (11.1%) in October 2009. We were not able to detect a seasonal pattern of influenza outbreaks in poultry (Figure 1); however, during our surveillance period, human cases of influenza (H5N1) virus infection were reported throughout the year and peaked in January and February 2009 (*8*) (Figure 1).

**Figure 2:**
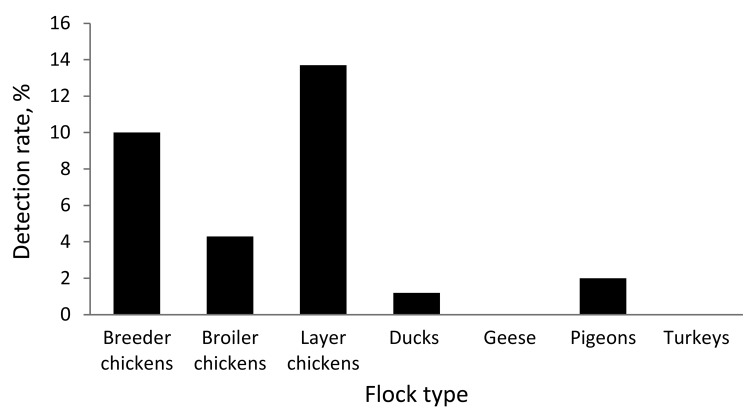
Rates of detection of influenza A (H5N1) virus by reverse transcription PCR, Egypt, August 2009–July 2010.

By species, ≈82% of the swab samples were collected from chickens, followed by ducks (14.7%) and other species of domestic birds (3.2%). Positivity rates differed significantly (p<0.001, Pearson χ^2^ test). Among chickens, 13.7% of the samples from layers, 10.0% from breeders, and 4.3% from broilers were positive. Among ducks, 1.2% of samples were positive. Only 1 pigeon swab sample was positive (Figure 2).

By collection location, the highest positivity rate (≈12%) came from poultry abattoirs (p<0.001, Pearson χ^2^ test). The next highest rates came from commercial farms (6.8%), followed by live-bird markets (3.3%). Only 0.9% of swab samples from backyard flocks were positive. Most (94.5%) samples were collected from apparently healthy birds; of those, 4.5% were positive. In contrast, 13.4% of samples from sick or dead birds were positive (p<0.001, Pearson χ^2^ test).

To identify other putative sources of human infection with influenza (H5N1) virus, we also examined a population of wild egrets (*Bubulcus ibis*) in urban greater Cairo. These birds congregate on trees next to the Giza Zoo in a heavy traffic area with a dense human population. RT-PCR detected influenza (H5N1) viruses in the feces of these wild egrets. Influenza (H5N1) virus shedding by the egrets threatens the exotic bird population at the zoo as well as humans in that area.

## Conclusions

In Egypt, most swab samples positive for influenza (H5N1) virus were from chickens. Among ducks, the positivity rate was as low as 1.2%, although in other regions, ducks have been shown to play a key role in avian influenza transmission (*9*). All samples with positive results by RT-PCR contained highly pathogenic influenza (H5N1) viruses. The surprising lack of detection of other influenza subtypes in our surveillance may be explained by establishment of subtype H5N1 as the dominant influenza strain in poultry in Egypt. Alternatively, low-pathogenicity viruses may be circulating in different regions or different host populations not covered by our surveillance. Whatever the reason, the lack of substantial cocirculation of multiple influenza viruses reduces the chances of influenza (H5N1) virus evolution occurring in Egypt by reassortment.

In Egypt, commercial farms are major reservoirs for influenza (H5N1) virus; the positivity rate was higher for those farms (7.2%) than for backyard farms (0.9%). Because the sampled poultry at commercial farms, where biosecurity measures were generally lax, were vaccinated with commercially available subtype H5 vaccines, the effectiveness of such vaccines becomes highly questionable. The lower positivity rate among backyard poultry may be explained by the fact that the growers slaughter these birds at the first sign of disease.

Reports of influenza (H5N1) virus infections in humans in Egypt show that most of these persons had had contact with sick poultry, primarily in backyards (*8**,**10*), as has been reported in Asia (*11**–**13*). Our findings indicate that the threat to humans in Egypt is much more widespread than previously reported. We detected influenza (H5N1) viruses in poultry from all production sectors and from wild egrets in Cairo. Among specimens collected from live-bird markets and slaughterhouses in Cairo, ≈6% had positive results; these birds usually come from commercial farms in rural areas. This finding indicates that the public health concern applies not only to rural poultry growers but also to persons in urban areas.

Although we were able to detect influenza virus among poultry continuously during surveillance, we did not establish a clear seasonal pattern of outbreaks, an indicator of continuous evolution of subtype H5N1 viruses endemic to Egypt. During the same period covered by our surveillance, human cases were reported during 8 of the 12 months; incidence was highest in January and February, reflecting a seasonal pattern conforming to the climate, with influenza activity peaks in the colder months. These data suggest that the seasonality of influenza (H5N1) in humans is not explained by increased virus activity in the associated poultry population but rather by other unidentified behavioral or environmental factors.

Our surveillance findings reveal that highly pathogenic influenza (H5N1) viruses are abundant and persistent in Egypt. Closer surveillance of avian influenza viruses in domestic poultry and expansion of such surveillance to include wild and migratory birds is warranted in an effort to continuously monitor the evolution of subtype H5N1 and other influenza viruses in Egypt.
